# Short-term intermittent hypoxia induces biphasic apoptotic responses in the murine heart

**DOI:** 10.1038/s41598-026-45151-7

**Published:** 2026-03-25

**Authors:** Semih Arbatli, Yüksel Peker

**Affiliations:** 1 Koc University Research Center for Translational Medicine (KUTTAM), Istanbul, Turkey; 2 Department of Pulmonary Medicine, Koc University School of Medicine, Istanbul, Turkey; 3 Department of Molecular and Clinical Medicine, Institute of Medicine, University of Gothenburg, Sahlgrenska Academy, Gothenburg, Sweden; 4 Department of Clinical Sciences, Respiratory Medicine and Allergology, Lund University School of Medicine, Lund, Sweden; 5 Division of Pulmonary, Allergy, and Critical Care Medicine, University of Pittsburgh School of Medicine, Pittsburgh, PA USA

**Keywords:** Intermittent hypoxia, Ischemic preconditioning, Cardiac apoptosis, Murine, Cardiology, Cell biology, Medical research, Physiology

## Abstract

Intermittent hypoxia (IH), the defining physiological feature of obstructive sleep apnea, has been strongly linked to cardiovascular morbidity, yet the early myocardial effects of short-term IH remain poorly characterized. In this study, we investigated whether brief IH exposure elicits adaptive anti-apoptotic signaling or early injurious activation of intrinsic apoptotic pathways in the murine heart. Adult male C57BL/6J mice were randomized into four groups (*n* = 8 per group): room air for 1 day, IH for 1 day, room air for 7 days, and IH for 7 days. IH consisted of alternating 30 s cycles of normoxia (21% O₂) and hypoxia (6% O₂) for 8 h/day for either one day or seven consecutive days. Cardiac tissues from the right ventricle, left ventricle, and interventricular septum were analyzed using histological assessment, apoptotic index quantification, and Western blot analysis of intrinsic apoptotic and hypoxia-related signaling pathways. One day of IH resulted in reduced apoptotic activity and increased expression of the anti-apoptotic protein Bcl-2, consistent with an early adaptive, preconditioning-like response. In contrast, seven days of IH markedly increased apoptosis, and was associated with elevated BAX, cleaved PARP, p53, and HIF-1α expression, indicating activation of intrinsic mitochondrial apoptotic pathways. A more pronounced effect was observed in the right ventricle. Fibrotic remodeling was minimal across all groups, suggesting that apoptosis precedes structural myocardial remodeling. These findings reveal a time-dependent transition from early anti-apoptotic adaptation to intrinsic apoptotic activation during short-term IH exposure and provide insight into the early myocardial consequences of intermittent hypoxia.

**Supplementary Information:**

The online version contains supplementary material available at 10.1038/s41598-026-45151-7.

## Introduction

Obstructive sleep apnea (OSA) is a prevalent sleep-related breathing disorder characterized by recurrent upper airway collapse during sleep, leading to repetitive episodes of intermittent hypoxia (IH), sleep fragmentation, and sympathetic activation. OSA is strongly associated with increased cardiovascular morbidity and mortality, including hypertension, coronary artery disease, arrhythmias, and heart failure^1–4^. Among the pathophysiological features of OSA, IH has emerged as a central mediator linking sleep-disordered breathing to adverse cardiovascular outcomes.

IH exposes the myocardium to repetitive hypoxia–reoxygenation cycles that promote oxidative stress, systemic inflammation, endothelial dysfunction, and autonomic imbalance^5–7^. Both experimental and clinical studies have shown that IH contributes to myocardial hypertrophy, ventricular dysfunction, and adverse cardiac remodeling, independent of traditional cardiovascular risk factors^8–10^. Despite this growing evidence, the early myocardial effects of short-term IH exposure remain poorly defined.

Cardiomyocyte apoptosis is a key mechanism underlying cardiac remodeling and functional deterioration. Excessive activation of apoptotic pathways leads to progressive loss of viable cardiomyocytes and contributes to ventricular dysfunction and heart failure^11,12^. In models of chronic IH, increased myocardial apoptosis has been consistently reported, involving mitochondrial signaling pathways regulated by the BAX/Bcl-2 balance, caspase activation, and downstream DNA damage responses^13–16^. These apoptotic changes are often accompanied by myocardial fibrosis and impaired cardiac function^17–19^.

Paradoxically, IH has also been reported to induce ischemic preconditioning–like cardioprotective effects under certain conditions. Short or early hypoxic exposure may activate adaptive pathways that suppress apoptosis and enhance cellular resilience, whereas prolonged exposure appears to overwhelm these mechanisms, resulting in maladaptive remodeling^20–22^. However, the temporal transition between adaptive and injurious myocardial responses to IH remains unclear. We therefore designed the present study to experimentally investigate whether short-term IHa could induce adaptive anti-apoptotic signaling resembling preconditioning, and whether such responses would persist or transition toward intrinsic apoptotic activation with continued exposure. We hypothesized that brief IH exposure may engage cardioprotective pathways, whereas sustained exposure would shift myocardial signaling toward apoptosis.

## Materials and methods

### Animals and ethical approval

Adult male C57BL/6J mice (8–10 weeks old, body weight 22–26 g) were used in this study. The animals were obtained from the Koc University Research Center for Translational Medicine (KUTTAM) animal facility and housed under controlled environmental conditions (22 ± 2 °C, 50–60% humidity) with a 12-h light/12-h dark cycle and had free access to standard laboratory chow and water. All experimental procedures were approved by the Koc University Animal Care and Use Ethics Committee (31/03/2023, approval nr 2022.HADYEK.022) and were conducted in accordance with the National Institutes of Health Guide for the Care and Use of Laboratory Animals.

#### Experimental design and intermittent hypoxia exposure

**Fig. 1:**
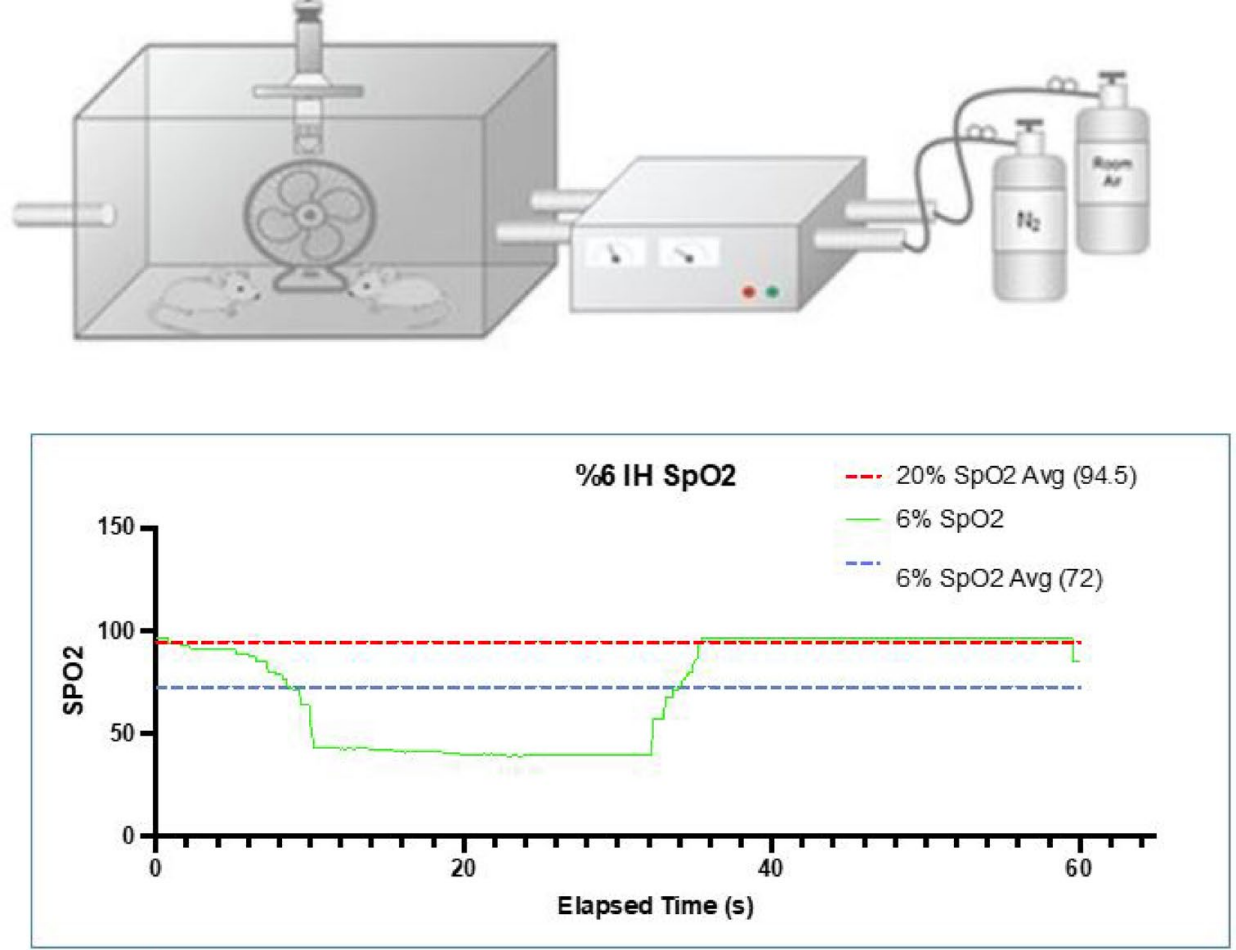
Intermittent hypoxia (IH) exposure protocol.

Mice were randomly assigned to four experimental groups (*n* = 8 per group):^1^ room air for 1 day (RA-1d)^2^, IH for 1 day (IH-1d)^3^, room air for 7 days (RA-7d), and^4^ IH for 7 days (IH-7d). Intermittent hypoxia was delivered using an automated gas-control chamber system for 8 h per day. Animals were randomly allocated to cages corresponding to each experimental condition prior to initiation of exposure protocols. Investigators performing histological quantification and Western blot analyses were blinded to group allocation during data acquisition and analysis. Each hypoxic cycle consisted of 30 s of normoxia (21% O₂) followed by 30 s of hypoxia (6% O₂), repeated continuously for either a single day (acute exposure) or seven consecutive days (subacute exposure). (Fig. 1). Control animals were housed in identical chambers under stable room air conditions and were exposed to the same environmental conditions without oxygen fluctuations.

Representative oxygen saturation (SpO₂) profile during intermittent hypoxia exposure is shown. Mice were exposed to alternating cycles of normoxia (21% O₂) and hypoxia (6% O₂) consisting of 30 s each, repeated continuously for 8 h/day. The experimental setup included an automated hypoxic chamber system, while control animals were maintained under stable room air conditions.

### Tissue collection and cardiac region separation

At the end of the exposure period, mice were anesthetized and euthanized according to approved protocols: C57BL/6 mice were anesthetized using isoflurane (2% for induction and 1.5% for maintenance), delivered via an inhalation chamber. Anesthesia was confirmed by the absence of a paw reflex. Once fully anesthetized, the mice were placed on a heated surface to maintain body temperature throughout the procedure. For blood collection, approximately 0.5 mL of blood was drawn via cardiac puncture. Following blood collection, the mice were euthanized by transcardial perfusion with cold phosphate-buffered saline (PBS). The perfusion was carried out using a peristaltic pump set to a flow rate of 4 mL/min. The perfusion was continued until the effluent from the right atrium was clear, indicating the removal of blood from the organs. Following perfusion, the heart was harvested for further analysis. Hearts were rapidly excised, rinsed in cold phosphate-buffered saline, and processed for histological and molecular analyses. The right ventricle (RV), left ventricle (LV), and interventricular septum (IVS) were identified and analyzed separately when applicable.

### Histological analysis and apoptotic index

Cardiac tissues were fixed in 4% paraformaldehyde, embedded in paraffin, and sectioned at 5 μm thickness. Sections were stained with hematoxylin and eosin (H&E) for morphological assessment.

**Fig. 2:**
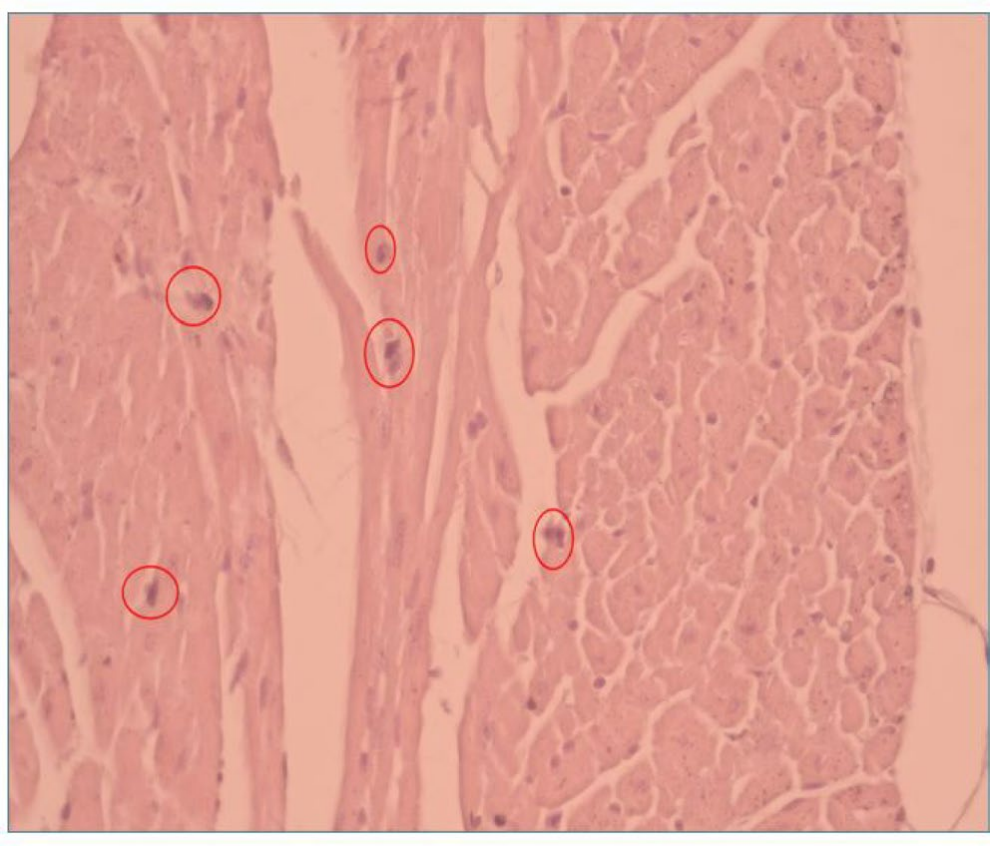
Identification of apoptotic cardiomyocytes in myocardial tissue.

Apoptotic activity was evaluated using an apoptotic index based on characteristic nuclear morphology, including chromatin condensation and nuclear fragmentation. For each animal, five non-overlapping high-power fields were analyzed per cardiac region by two independent investigators blinded to group allocation. The apoptotic index was calculated as the percentage of apoptotic nuclei relative to the total number of nuclei (Fig. 2).

Representative hematoxylin and eosin (H&E)–stained myocardial section illustrating apoptotic cardiomyocytes characterized by nuclear condensation and fragmentation. Apoptotic cells are indicated by circles.

### Assessment of myocardial fibrosis

To evaluate collagen deposition, selected sections were stained using Masson’s trichrome. Fibrotic remodeling was assessed qualitatively by examining interstitial and perivascular collagen accumulation across experimental groups.

### Western blot analysis

Protein extracts from cardiac tissues were prepared using standard lysis buffers supplemented with protease inhibitors. Equal amounts of protein were separated by SDS–PAGE and transferred onto PVDF membranes. Membranes were incubated with primary antibodies against BAX, Bcl-2, cleaved poly(ADP-ribose) polymerase (PARP), hypoxia-inducible factor-1α (HIF-1α), and p53, followed by appropriate horseradish peroxidase–conjugated secondary antibodies. Immunoreactive bands were visualized using enhanced chemiluminescence and quantified by densitometry. Protein expression levels were normalized to housekeeping proteins and expressed relative to room air controls.

### Statistical analysis

Data are presented as box plots showing median and interquartile range. Statistical analyses were performed using appropriate parametric or non-parametric tests depending on data distribution. Comparisons were made between intermittent hypoxia and corresponding room air control groups, as well as between one-day and seven-day IH exposure. A two-sided p value < 0.05 was considered statistically significant. All analyses were conducted with investigators blinded to experimental groups.

## Results

### Intermittent hypoxia induces a time-dependent biphasic apoptotic response in the murine heart

All animals tolerated intermittent hypoxia (IH) exposure without mortality or overt signs of distress. Oxygen modulation within the hypoxia chamber produced stable and reproducible hypoxia–reoxygenation cycles throughout the exposure period, as illustrated by representative saturation profiles and experimental schematics (Fig. 1).

**Fig. 3:**
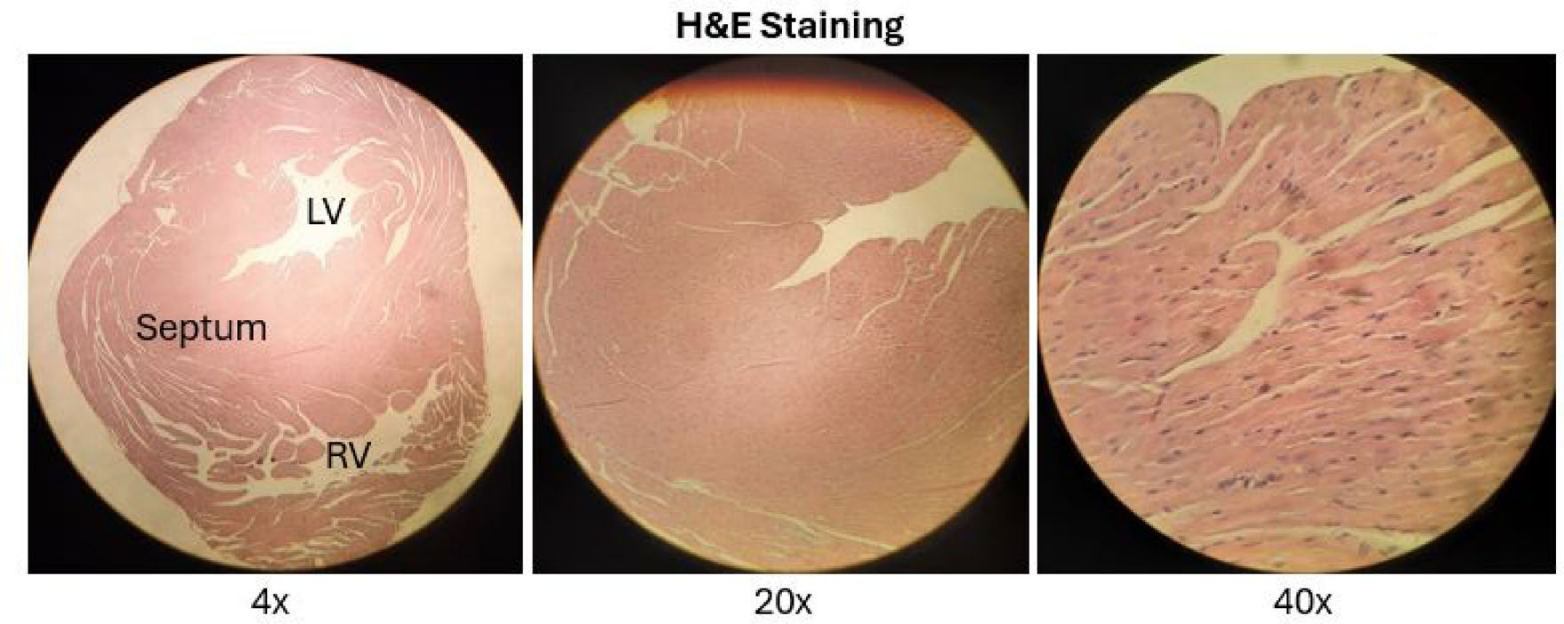
Representative myocardial histology and regional anatomy.

Histological examination revealed a clear time-dependent biphasic apoptotic response to IH. After one day of IH exposure, myocardial sections demonstrated preserved cellular architecture with a reduction in apoptotic nuclear morphology compared with room air controls across all examined cardiac regions, including the right ventricle (RV), left ventricle (LV), and interventricular septum (IVS) (Fig. 3).

Representative hematoxylin and eosin (H&E)–stained sections of murine heart tissue shown at low (4×) and higher magnifications (20× and 40×), illustrating overall myocardial architecture and regional organization of the left ventricle (LV), interventricular septum, and right ventricle (RV).

**Fig. 4:**
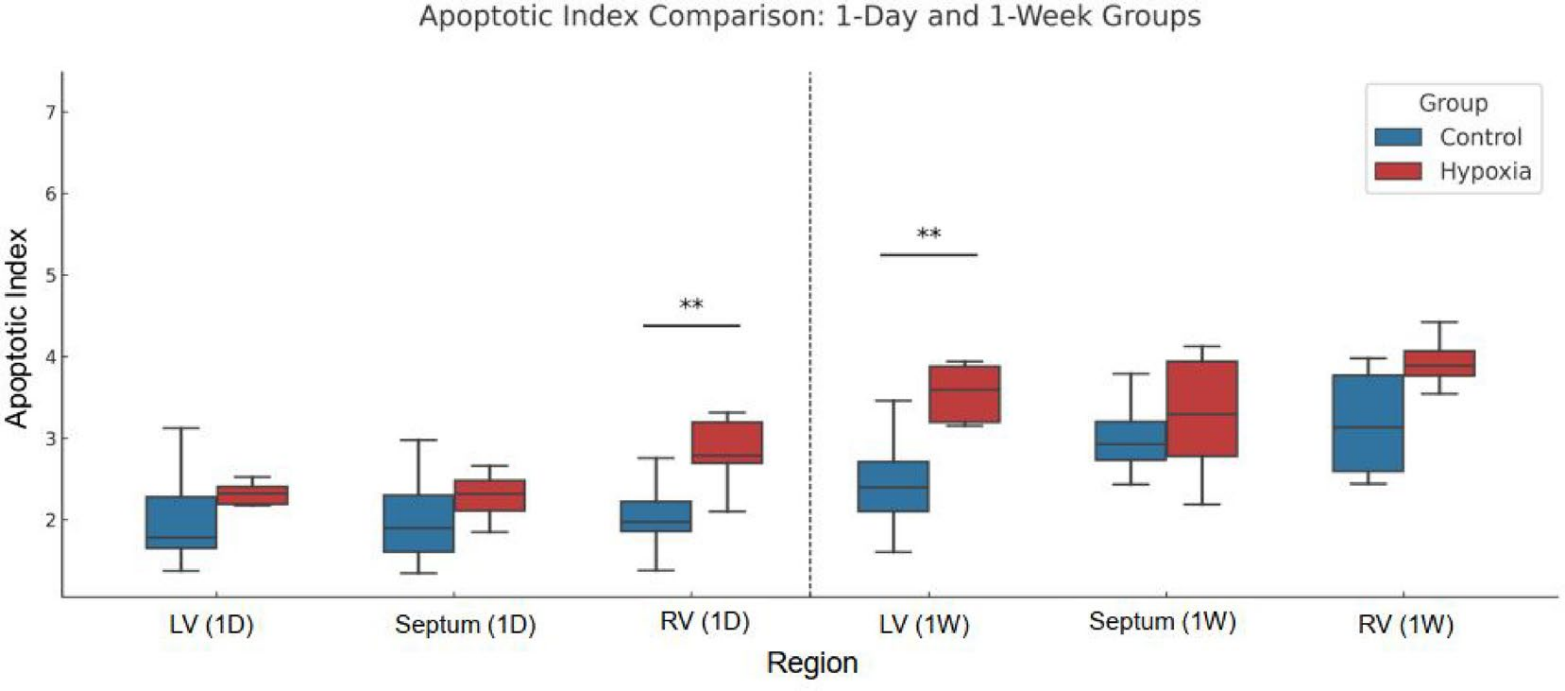
Quantification of myocardial apoptotic index following intermittent hypoxia.

As illustrated in Fig. 4, quantification of the apoptotic index confirmed a significant decrease following one day of IH. In contrast, seven days of IH exposure resulted in a marked increase in apoptotic nuclei compared with both the corresponding room air control group and the one-day IH group. This increase was most pronounced in the RV, whereas the LV and IVS exhibited more moderate but consistent changes (Fig. 4). These findings indicate a transition from early suppression of apoptosis to regionally dominant apoptotic activation with sustained IH exposure.

Apoptotic index was quantified in the left ventricle (LV), interventricular septum, and right ventricle (RV) after 1 day and 7 days of intermittent hypoxia exposure. Data are presented as box plots showing median and interquartile range. Statistical comparisons were performed between intermittent hypoxia and corresponding room air control groups.

### Differential regulation of apoptotic signaling pathways by short-term intermittent hypoxia

**Fig. 5:**
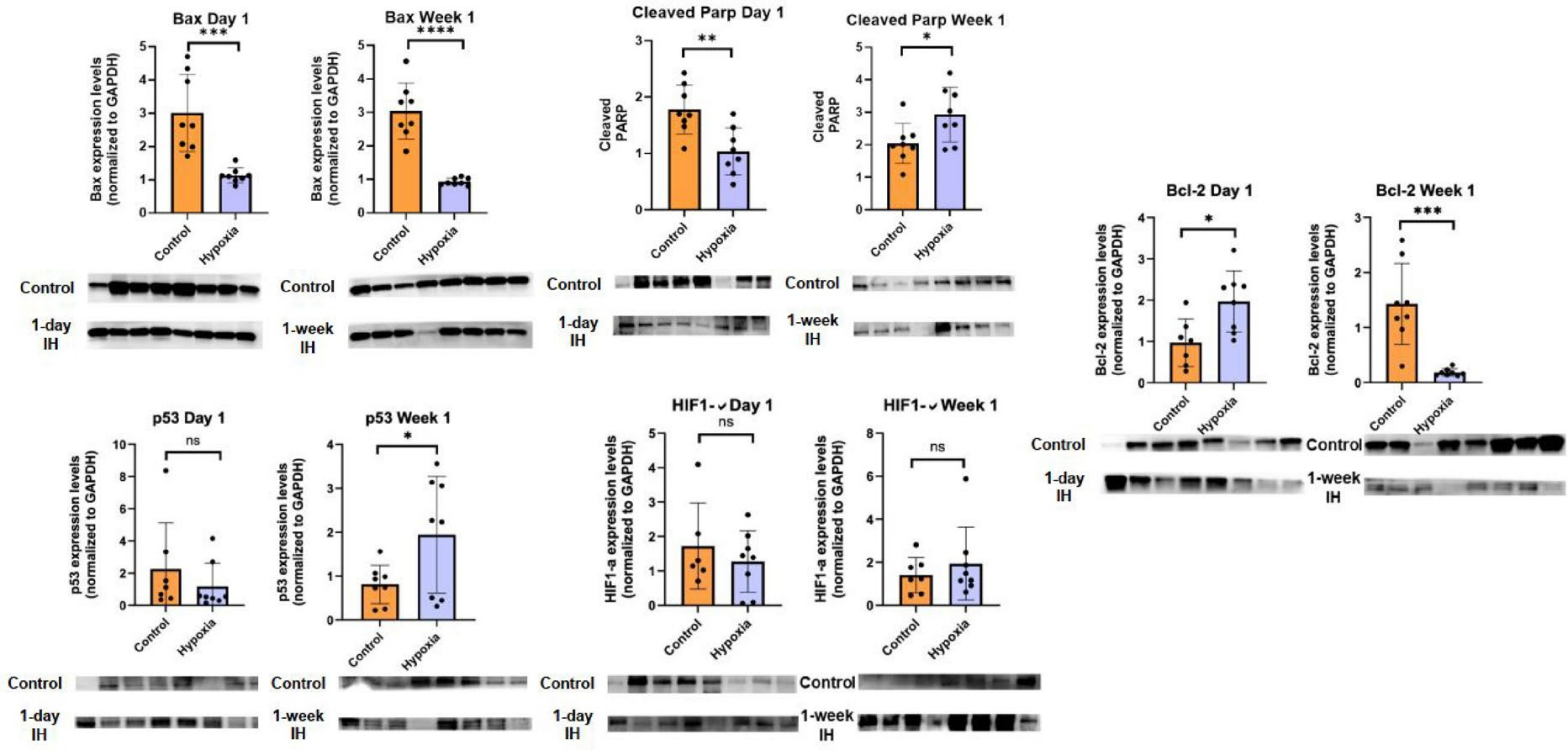
Regulation of apoptotic and hypoxia-related signaling pathways following intermittent hypoxia.

As illustrated in Fig. 5, western blot analysis supported the histological findings and demonstrated time-dependent regulation of apoptotic signaling pathways. Following one day of IH exposure, expression of the anti-apoptotic protein Bcl-2 was increased, while expression of the pro-apoptotic protein BAX was reduced, resulting in a decreased BAX/Bcl-2 ratio compared with room air controls. Moreover, levels of cleaved poly (ADP-ribose) polymerase (PARP) were reduced after one day of IH.

Representative Western blot images and quantitative densitometric analyses of BAX, cleaved PARP, p53, and HIF-1α (pro-apoptotic and stress-related markers), together with the anti-apoptotic protein Bcl-2, in myocardial tissue following 1 day and 7 days of intermittent hypoxia exposure. Protein expression levels were normalized to GAPDH and expressed relative to corresponding room air control groups. Original, unprocessed blot segments corresponding to these images are provided in Supplementary Figures S1 (intermittent hypoxia groups) and S2 (control groups). Membranes were cut according to molecular weight ranges prior to antibody incubation.

After seven days of IH exposure, a reversal of this pattern was observed. BAX expression was significantly increased, whereas Bcl-2 expression was reduced, leading to an increased BAX/Bcl-2 ratio (Fig. 5). Cleaved PARP levels were markedly elevated, indicating activation of intrinsic apoptotic pathways. In parallel, expression of hypoxia-inducible factor-1α (HIF-1α) and p53 was increased following seven days of IH exposure, with the most prominent changes observed in the RV.

### Minimal fibrotic remodeling following short-term intermittent hypoxia

**Fig. 6:**
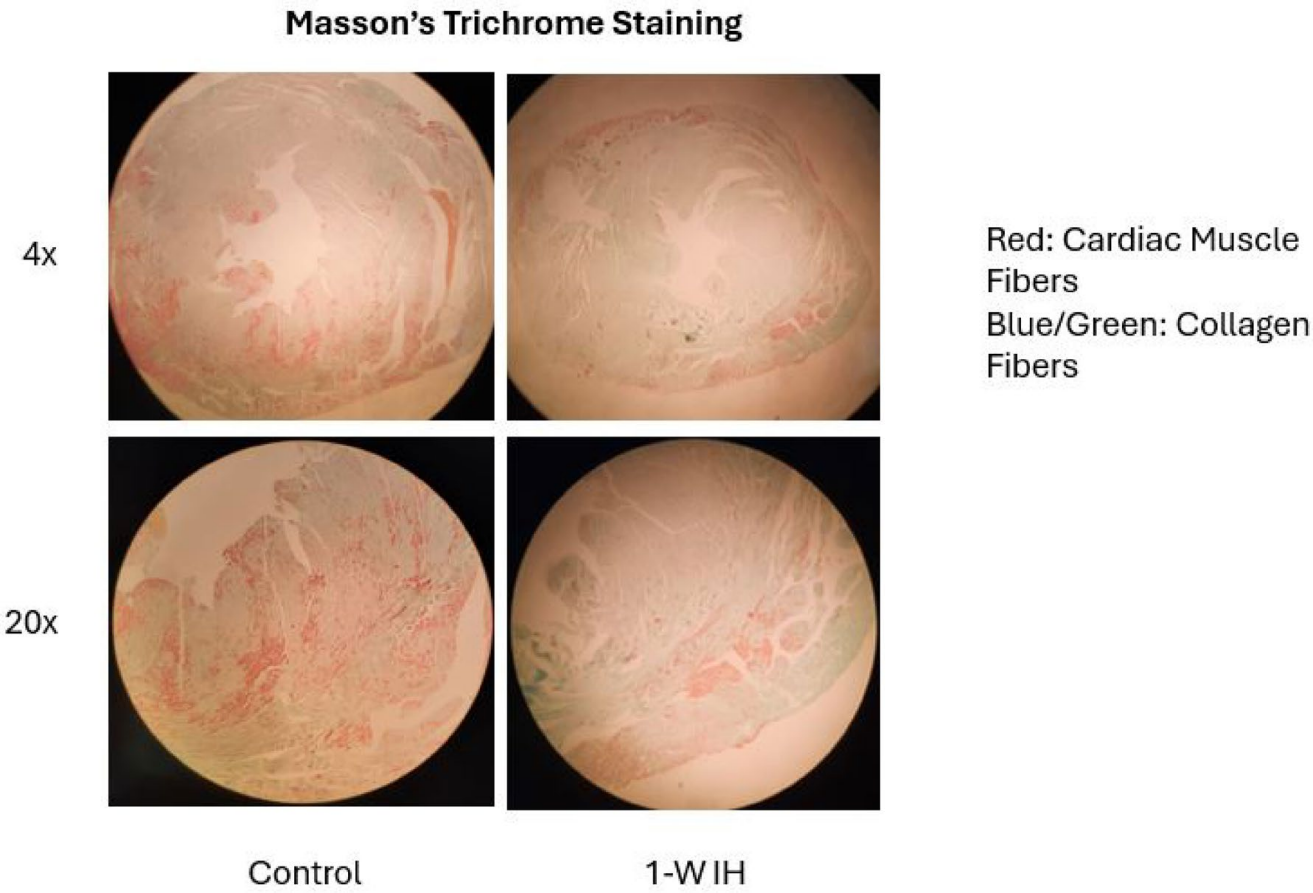
Assessment of myocardial fibrosis following short-term intermittent hypoxia.

Despite the observed changes in apoptotic signaling, myocardial architecture remained largely preserved. Hematoxylin and eosin staining showed no evidence of necrosis or inflammatory cell infiltration in any experimental group. Assessment of collagen deposition using Masson’s trichrome staining revealed minimal interstitial or perivascular fibrosis following both one and seven days of IH exposure, with no appreciable differences compared with room air controls (Fig. 6).

Representative Masson’s trichrome–stained sections of myocardial tissue from room air control and 7-day intermittent hypoxia–exposed mice. Cardiac muscle fibers are shown in red, whereas collagen deposition is indicated in blue/green. No overt fibrotic remodeling was observed following short-term intermittent hypoxia.

## Discussion

### Principal findings

The present study demonstrates that short-term intermittent hypoxia (IH) induces a time-dependent biphasic apoptotic response in the murine heart. One day of IH exposure was associated with suppression of myocardial apoptosis and enhanced anti-apoptotic signaling, whereas seven days of IH resulted in a clear shift toward intrinsic apoptotic activation, particularly in the right ventricle. Importantly, these changes occurred in the absence of overt fibrosis, indicating that apoptosis represents an early and potentially reversible stage of IH-induced myocardial injury.

### Intermittent hypoxia as a preconditioning-like stimulus

A key finding of this study is that very short-term IH exposure reduced myocardial apoptotic activity, a pattern consistent with ischemic preconditioning–like cardioprotection. Ischemic preconditioning describes the phenomenon whereby brief, non-lethal ischemic or hypoxic episodes activate adaptive cellular pathways that enhance myocardial tolerance to subsequent injury A central conceptual motivation for this study was the clinical and experimental observation that IH may, under specific conditions, resemble ischemic preconditioning. Ischemic preconditioning is characterized by activation of mitochondrial stabilization pathways, increased expression of anti-apoptotic proteins, and suppression of caspase-mediated cell death following brief, non-lethal stress^23–25^. Classical ischemic preconditioning involves activation of pro-survival signaling cascades, including PI3K/Akt, ERK1/2, mitochondrial KATP channels, and redox-sensitive transcriptional programs^26–28^. Although these pathways were not directly assessed in the present study, the observed increase in Bcl-2 expression and reduction in PARP cleavage after 1 day of IH are consistent with activation of mitochondrial stabilization mechanisms typically observed in preconditioning models. Experimental models have shown that short hypoxic exposures can suppress apoptosis, stabilize mitochondrial membranes, and activate pro-survival signaling cascades, including hypoxia-inducible factor-1α (HIF-1α)–dependent pathways^29–31^().

In the present study, one day of IH exposure resulted in increased Bcl-2 expression and reduced PARP cleavage, consistent with engagement of anti-apoptotic mitochondrial regulatory mechanisms. These findings support the concept that early IH may transiently activate adaptive cardioprotective signaling. Importantly, this adaptive phenotype did not persist with continued exposure. After seven days, pro-apoptotic signaling predominated, suggesting that sustained hypoxic burden overwhelms early protective pathways and promotes intrinsic mitochondrial apoptosis. Similar preconditioning-like effects of intermittent hypoxia have been reported in animal models, where brief IH exposure reduced infarct size and limited cardiomyocyte loss following ischemic challenge^17,32,33^. Together, these findings suggest that short-term IH may transiently engage endogenous cardioprotective mechanisms. Importantly, this adaptive phenotype did not persist with continued exposure. After seven days, pro-apoptotic signaling predominated, suggesting that sustained hypoxic burden overwhelms early protective pathways and promotes intrinsic mitochondrial apoptosis.

### Transition from adaptation to injury with sustained IH

In contrast to the early adaptive response, seven days of IH exposure resulted in marked activation of intrinsic apoptotic signaling, including increased BAX expression, PARP cleavage, p53 activation, and upregulation of HIF-1α. This shift likely reflects exhaustion of preconditioning mechanisms and transition toward maladaptive remodeling. Prolonged or repetitive IH has been shown to promote mitochondrial dysfunction, oxidative stress, and sustained activation of apoptotic pathways, ultimately leading to cardiomyocyte loss^34–36^. Beyond HIF-1α, intermittent hypoxia is known to activate multiple redox- and metabolism-sensitive signaling pathways that contribute to both adaptive and maladaptive responses. In particular, NF-κB–mediated inflammatory signaling, Nrf2-dependent antioxidant transcriptional programs, and AMP-activated protein kinase (AMPK)–regulated metabolic adaptation have been implicated in cellular responses to intermittent hypoxia^37^. NF-κB activation may promote inflammatory and pro-apoptotic signaling under sustained hypoxic stress, whereas Nrf2 induction and AMPK activation can enhance antioxidant defenses and metabolic resilience during early adaptive phases. Although these pathways were not directly assessed in the present study, they likely interact with the observed mitochondrial apoptotic signaling cascade and may contribute to the temporal transition from early anti-apoptotic adaptation to later intrinsic apoptotic activation.

These observations align with the concept that the cardioprotective effects of preconditioning are highly time- and dose-dependent, and that persistent hypoxic stress overwhelms adaptive signaling^38,39^. Thus, the duration of IH exposure emerges as a critical determinant of whether myocardial responses remain adaptive or become injurious.

### Regional vulnerability of the right ventricle

The right ventricle exhibited the most pronounced apoptotic response following seven days of IH exposure. Although the study was not specifically designed to test regional susceptibility, this observation emerged consistently across histological and molecular analyses. This regional vulnerability likely reflects the unique structural and functional characteristics of the right ventricle, including thinner myocardial walls, lower perfusion reserve, and heightened sensitivity to increases in pulmonary vascular resistance^40,41^. Intermittent hypoxia induces transient pulmonary vasoconstriction, thereby increasing right ventricular afterload and mechanical stress^42,43^. Clinical studies in patients with obstructive sleep apnea similarly demonstrate early right ventricular dysfunction and remodeling, even in the absence of left ventricular involvement^40–42,44–46^. The present findings provide mechanistic insight into these observations by identifying early, region-specific apoptotic activation.

### Apoptosis precedes fibrotic remodeling

Despite robust apoptotic signaling after seven days of IH exposure, myocardial fibrosis remained minimal. This dissociation supports the concept that apoptosis precedes extracellular matrix remodeling in the early stages of IH-induced cardiac injury. Experimental studies indicate that sustained cardiomyocyte apoptosis initiates fibroblast activation and collagen deposition over longer time frames^47–49^. The absence of fibrosis in the present study underscores the existence of a potential therapeutic window during which modulation of apoptotic pathways may prevent irreversible myocardial remodeling.

### Implications for OSA-related cardiovascular disease

These findings have important implications for cardiovascular risk in obstructive sleep apnea. Clinical data increasingly suggest that cardiovascular outcomes in OSA are determined not solely by apnea frequency but by the burden and chronicity of intermittent hypoxia^50–52^( The preconditioning-like effects observed with very short-term IH may help explain why early or mild OSA does not uniformly translate into cardiac injury, whereas sustained hypoxic exposure confers substantial cardiovascular risk. Moreover, these data may offer mechanistic insight into the heterogeneous cardiovascular responses observed in clinical trials of continuous positive airway pressure therapy, where benefits appear confined to specific patient subgroups^53,54^.

### Strengths and limitations

The strengths of this study include a controlled IH model, precise temporal comparison of short-term exposures, region-specific cardiac analysis, and integration of histological and molecular endpoints. Limitations include the exclusive use of male mice, absence of functional cardiac assessments, relatively short exposure duration, and lack of comprehensive upstream signaling analysis. Specifically, we did not directly evaluate NF-κB activation, Nrf2 signaling, antioxidant enzyme activity, or AMPK phosphorylation, which are known modulators of intermittent hypoxia–induced redox and metabolic responses. Future studies incorporating integrated pathway analysis will be necessary to fully define the signaling network underlying IH-induced myocardial remodeling.

## Conclusions

In conclusion, this study demonstrates that intermittent hypoxia exerts dual, time-dependent effects on the murine myocardium. Short-term exposure induces a preconditioning-like, anti-apoptotic response, whereas sustained exposure promotes activation of intrinsic apoptotic pathways, with a more pronounced effect observed in the right ventricle. These findings refine current understanding of IH-induced cardiac injury and highlight the importance of exposure duration in determining myocardial outcome. Targeting early apoptotic signaling pathways may represent a promising strategy to prevent progression of cardiac injury in disorders characterized by intermittent hypoxia, such as OSA.

## Supplementary Information

Below is the link to the electronic supplementary material.

Supplementary Material 1

## Data Availability

The datasets generated and/or analysed during the current study are not publicly available because they include primary experimental data and raw imaging files (histological and immunoblot images) obtained from animal experiments and are subject to ethical and institutional data-sharing restrictions, but are available from the corresponding author on reasonable request.
