# Supplementary material for: Short-term intermittent hypoxia induces biphasic apoptotic responses in the murine heart
Source: Sci Rep. 2026 Mar 25;16:14974. doi: 10.1038/s41598-026-45151-7 (PMC13172486; doi:10.1038/s41598-026-45151-7)
Supplement: Supplementary file 1 — Supplementary Material 1 [file 41598_2026_45151_MOESM1_ESM.pdf]

Supplementary Figure S1. Full-length uncropped Western blot images from the intermittent hypoxia groups.

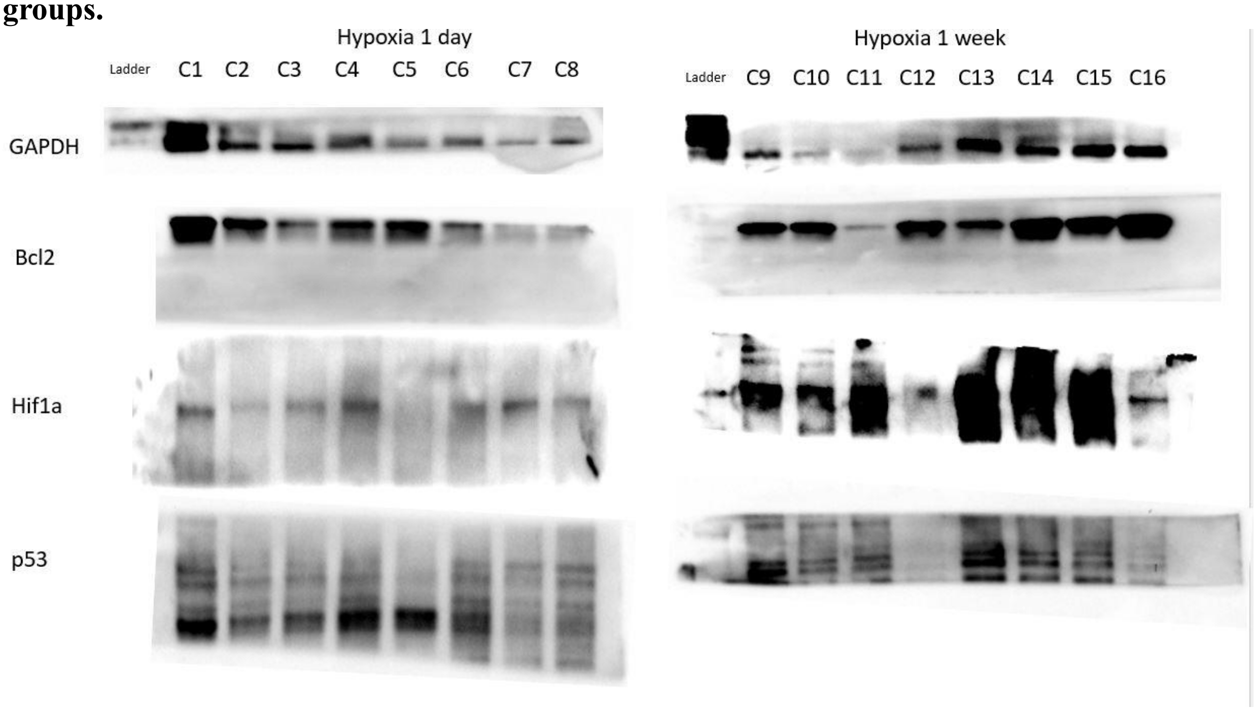

Full-length, uncropped Western blot images corresponding to the cropped blots shown in Figure 5 for the intermittent hypoxia groups (1 day and 1 week) are presented. Lanes represent individual samples run on the same or comparable gels under identical experimental conditions. Contrast adjustments were applied uniformly across entire images.

Supplementary Figure S2. Full-length uncropped Western blot images from the control groups.

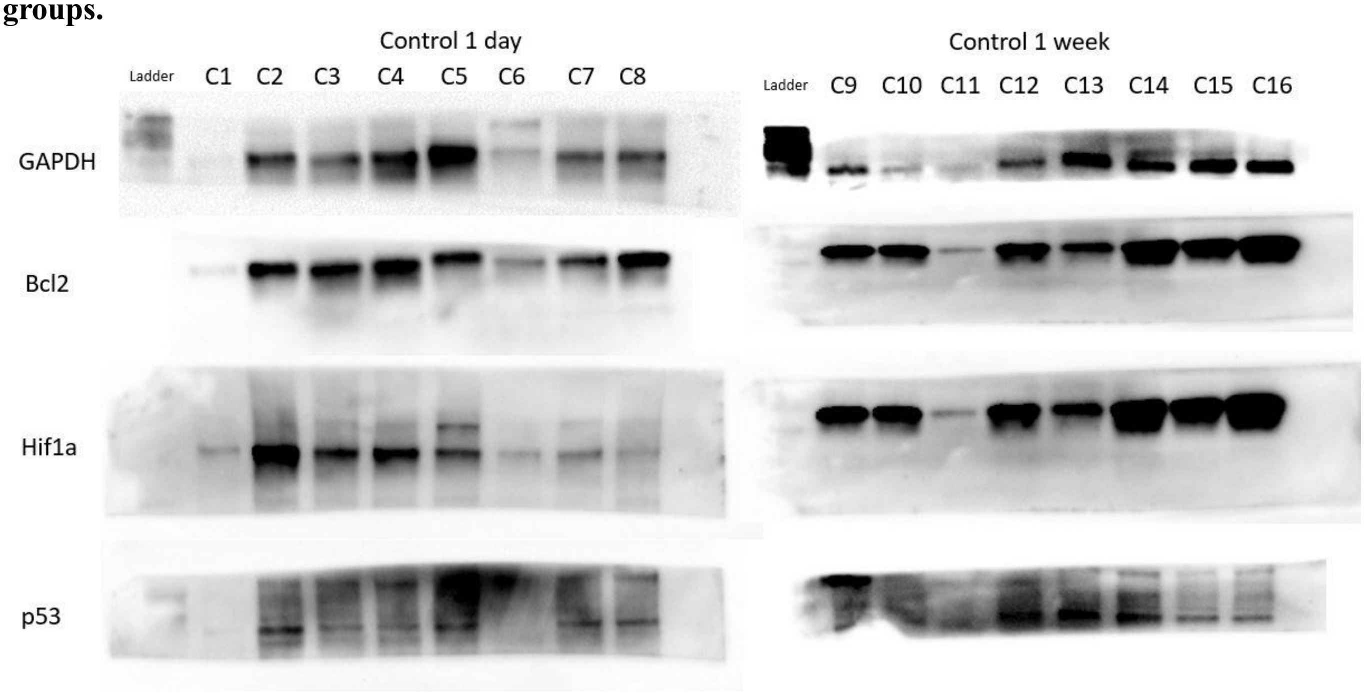

Full-length, uncropped Western blot images corresponding to the cropped blots shown in Figure 5 for the room air control groups (1 day and 1 week) are presented. Lanes represent individual samples run on the same or comparable gels under identical experimental conditions. Contrast adjustments were applied uniformly across entire images.
